# Selective Vulnerability Related to Aging in Large-Scale Resting Brain Networks

**DOI:** 10.1371/journal.pone.0108807

**Published:** 2014-10-01

**Authors:** Hong-Ying Zhang, Wen-Xin Chen, Yun Jiao, Yao Xu, Xiang-Rong Zhang, Jing-Tao Wu

**Affiliations:** 1 Department of Radiology, Subei People's Hospital of Jiangsu Province, Yangzhou University, Yangzhou, China; 2 Jiangsu Key Laboratory of Molecular and Functional Imaging, Southeast University, Nanjing, China; 3 Department of Neurology, Subei People's Hospital of Jiangsu Province, Yangzhou University, Yangzhou, China; 4 Department of Psychiatry, Zhongda Hospital, Southeast University, Nanjing, China; National Scientific and Technical Research Council (CONICET), Argentina

## Abstract

Normal aging is associated with cognitive decline. Evidence indicates that large-scale brain networks are affected by aging; however, it has not been established whether aging has equivalent effects on specific large-scale networks. In the present study, 40 healthy subjects including 22 older (aged 60–80 years) and 18 younger (aged 22–33 years) adults underwent resting-state functional MRI scanning. Four canonical resting-state networks, including the default mode network (DMN), executive control network (ECN), dorsal attention network (DAN) and salience network, were extracted, and the functional connectivities in these canonical networks were compared between the younger and older groups. We found distinct, disruptive alterations present in the large-scale aging-related resting brain networks: the ECN was affected the most, followed by the DAN. However, the DMN and salience networks showed limited functional connectivity disruption. The visual network served as a control and was similarly preserved in both groups. Our findings suggest that the aged brain is characterized by selective vulnerability in large-scale brain networks. These results could help improve our understanding of the mechanism of degeneration in the aging brain. Additional work is warranted to determine whether selective alterations in the intrinsic networks are related to impairments in behavioral performance.

## Introduction

The decline of functions such as memory, attention, problem-solving and sensorimotor ability is commonly experienced in old age. Such age-related cognitive decline may involve the selective deterioration of specific brain systems. Many hypotheses have been proposed to describe the aging process of the brain [1], [2]. Studying the aged brain with respect to the networks that sustain brain functions may be helpful in understanding the complicated, age-related changes that occur in this organ.

Measuring synchronic low-frequency fluctuations (LFFs) among spatially distant brain regions using blood oxygen level-dependent (BOLD) functional magnetic resonance imaging (fMRI) signals is useful for mapping intrinsic neural networks in healthy and diseased subjects. Researchers have identified resting state neural network (RSN) architectures that are presumed to underlie the higher-order cognitive functions that are associated with memory, attention, execution and emotion processing [3]–[6] in addition to motor and sensory functions [7], [8]. The corresponding higher-order networks include the following: 1) a default mode network (DMN), 2) a dorsal attention network (DAN), 3) an executive control network (ECN) and 4) a salience network (SN). The DMN is involved in internal and external environment alerts as well as memory- and self-related functions, and it presents temporospatial anti-correlations with other canonical networks during externalized cognitive tasks [5], [9]–[11]. These canonical networks have been systemically measured in patients with neuropsychiatric disorders and have shown selective vulnerability in distinct neurodegenerative illnesses, such as Alzheimer's disease (AD) and frontotemporal dementia syndromes [12]–[15]. For instance, the DMN selectively disrupts the connectivity in patients with AD but enhances the connectivity in patients with behavioral variant frontotemporal dementia, whereas the SN shows enhanced activities in patients with AD and disrupted connectivity in patients with behavioral variant frontotemporal dementia [13]. These canonical networks are also differentially affected in schizophrenic patients compared to controls; schizophrenic patients demonstrate increased connectivity in the DMN, less connectivity in the ECN and DAN, and no difference in the salience network [16]. These findings indicate that the selective alterations in large-scale resting brain networks could represent a characteristic of neuropsychiatric disorders.

Aging, which could broadly affect multiple brain systems, is considered to be the primary risk factor for neurodegenerative disorders, such as sporadic AD. Numerous conventional task-based and resting-state fMRI investigations have been conducted to understand the effects of aging on brain function. In task-based studies, abnormally decreased or increased brain activation was found in normal elderly subjects during various cognitive processes, such as memory, attention and semantic judgments [17]–[19]. The alterations in the balance between DMN and task-related activity could account for the increased vulnerability to distraction by irrelevant information among the elderly subjects [17]. Additional investigations revealed that aging is associated with changes in LFF correlations between different cortical regions during task performance and in the resting state [20]–[25]. Stronger resting-state network connectivity in older adults is associated with better performance in tests of executive function and processing speed, such as the Trail Making Test, the word frequency task and face-name associative memory task [20], [24], [25]. Accumulating evidence from neuroimaging studies has suggested that the decline in cognitive functions is accompanied by focal changes in activity in several brain regions, such as the prefrontal cortex [26]–[28], hippocampal formation [28]–[30], and parietal regions [19], [27]. The evidence from graph theoretical analyses has indicated that older adults have decreased topological efficiency in small-world and modular organization of the entire brain [22], [31], [32]. In the present study, we investigated how the four canonical intrinsic networks underpinning cognitive functions change with aging.

Many studies have strongly indicated that resting-state functional connectivity provides relevant information regarding the effects of aging on brain functioning and cognition (for a review, see [33]). The most consistent age-related change is decreased resting-state connectivity in the DMN, which is crucial for memory [20]–[23], [32], [34]; another convergent finding is that the DAN is affected by the aging process [28], [32], [35]. Aging has a particular effect on the hub-related regions in the brain. The patterns of low-level anti-correlations between the DMN sub-networks and task-positive networks have also been demonstrated [23]. The findings noted from the topological analyses consistently suggest that the long-range connections mainly within the DMN and DAN regions are more vulnerable to aging effects compared with the short-range connections [31], [32], [36].

Age has a strong impact on the DMN throughout an individual's lifespan, and age-related changes in interregional functional connectivity exhibit spatially and temporally specific patterns [36], [37]. In a broad spectrum of populations, resting-state fMRI studies have shown reliable and replicable results [38]–[40]. Particularly in healthy older adults, such reliability has been supported by a recent test-retest study that evaluated different methods for data processing, including seed-based, independent component analyses and graph theoretical approaches [41].

A majority of the studies have focused on the aging-related DMN and DAN, simultaneously ignoring several specified high-order networks. The ECN, which includes the anterior prefrontal cortex, insular and frontal opercular cortices, and the dorsal anterior cingulated cortex [11], is modulated by dopamine and is functionally involved in cognitive control, attention shift control and decision-making [11], [42]. The behavioral theories of cognitive aging suggest that age-related decline in a range of cognitive tasks is mediated by selective impairment in executive processing functions [43]–[45]. Using neurobehavioral tests, such as an attention network test, previous studies have indicated that the executive effect is more significantly decreased with age than other functions, such as the alerting function and orienting attention function [46]. Although this observation indicates the existence of selective cognitive differences with advanced age, corresponding observations from neuroimaging are still required. Diffusion tensor MRI research provides a structural basis for the selective loss of executive functions by measuring the correlations between the changes in the frontal white matter integrity and the executive function performance assessed using the Trail Making Test [47]. A previous study demonstrated that moderate exercise attenuates the disruption of age-related resting brain connectivity between the regions in the ECN and in the DMN [48]. Although there have been a few examinations of age-related disruption in intrinsic functional connectivity in the executive control and salience networks [47]–[49], much of the literature supports age-related decline in the performance of tasks implanted in the ECN (for reviews, see [33], [45], [50]).

Several studies have measured topological whole-brain connectivity by applying the graph theory, which demonstrated significant decreases in intermodular connections to the frontal modular regions in older groups and revealed that normal aging is associated with the functional segregation of large-scale brain systems [22], [31], [37]. One study indicated that aging impacts not only connectivity within networks but also connectivity between different functional networks [51]. Indeed, a specific brain network may have more relevance to a specific function than to other functions. However, whether aging has equivalent effects or selective effects on specific large-scale networks has not been established. Thus, the purpose of our study was to investigate the effects of aging on four canonical RSNs including the DMN, DAN, ECN and SN by comparing two groups consisting of younger and older adults. We hypothesize that the alterations in the canonical networks would vary across the networks; therefore, we are especially concerned with the degree to which the large-scale intrinsic networks are affected. Because previous studies have shown that reduced functional connectivity in the motor network is related to aging [52], we used the visual system network as a control.

## Materials and Methods

### Subjects and procedure

This study was approved by the Yangzhou University Research Ethics Committee. All the participants provided their written informed consent.

A total of 40 right-handed healthy subjects were recruited, including 18 younger subjects (age, 22–33 years; mean age, 23.9±1.8 years; n = 9 males, 9 females) and 22 older adults (age, 60–80 years; mean age, 69.8±5.8 years; n = 10 males, 12 females). Both groups were education- and gender-matched. None of the subjects had a history of head trauma, neuropsychiatric disorders, hypertensive disease or metabolic disorders. Additionally, none of the subjects showed abnormal findings in their structural brain MRIs. Each subject had a Mini-Mental State Examination (MMSE) score ranging between 28 and 30.

Imaging was performed using a 1.5T GE Signa Excite MR scanner (GE Healthcare Systems, Milwaukee, WI, USA) and a standard head coil. Before the fMRI scan, the subjects were instructed to lie quietly with their eyes closed and to avoid thinking of anything in particular. The functional images were obtained using a gradient echo-planar imaging (EPI) sequence (TR, 3000 ms; TE, 40 ms; flip angle, 90°; slice thickness, 6 mm; slice gap, 0 mm; FOV, 240 mm; and matrix, 64×64), and each frame included 18 contiguous slices that covered the entire cerebral volume. The slices were obtained parallel to the anterior/posterior commissures. The EPI scan lasted 6 min and 24 sec. Finally, a T1-weighted 3D fast spoiled gradient echo sequence was acquired (TR, 70 ms; TE, 4.2 ms; FOV, 240 mm; matrix, 256×256; slice thickness, 1 mm).

### Data preprocessing and analysis

The fMRI data preprocessing was performed using DPARSF software V2.3 (http://www.restfmri.net), which is based on SPM8 (http://www.fil.ion.ucl.ac.uk/spm) and the Resting-State fMRI Data Analysis Toolkit (Beijing Normal University, Beijing, http://www.restfmri.net). The first four volumes were discarded. The images were corrected for slice timing and realigned for head movement correction. One young subject and two elderly subjects who failed to meet the study criterion were excluded because their head translation exceeded 1.5 mm or their head rotation exceeded 1.5°. There was no statistical significance between the groups for the movement parameters (two sample *t* test; the *p* values were 0.92, 0.93, and 0.55 for maximum, squared, and framewise displacement of the translation, respectively, and 0.60, 0.21, 0.12 for maximum, squared, and framewise displacement of the rotation, respectively). The functional images were normalized using DARTEL. The normalized volumes were re-sampled to a voxel size of 3 mm × 3 mm × 3 mm in MNI space, and the EPI images were spatially smoothed using an isotropic Gaussian filter (8 mm FWHM). The movement parameters (Friston 24-parameter model [53]) and signals of white matter, cerebrospinal fluid and global mean were regressed out. Linear detrending and temporal bandpass filtering (0.01–0.08 Hz) were applied.

The subsequent data processing and statistical analyses were performed using the Resting state fMRI data Analysis Toolkit (REST V1.8) (http://www.restfmri.net). For connectivity analysis, we created eight seed regions of interest (ROIs) with 6-mm radius spheres, which have been used in previous studies to identify the corresponding RSNs [11], [16], [54]. Each RSN and the seed ROI (in MNI coordinates) were as follows: DMN (PCC: −2, −54, 27; ventral medial prefrontal cortex: −2, 55, 6); ECN (left and right dorsolateral prefrontal cortex (dLPFC): −42, 34, 20/44, 36, 20); DAN (left and right superior parietal lobule (SPL): −25, −53, 52/25, −57, 52); and SN (left and right frontal-insular cortex (FI): −32, 26, −14/38, 22, −10). The positive Pearson correlation coefficients between the time series of each ROI and the time series in other voxels in the brain were calculated. A Fisher's z-transform was applied to improve the normality of these correlation coefficients. For the DMN, connectivity maps derived from the anterior and posterior seeds were averaged to create a single connectivity map; for the other canonical networks with bilateral ROI seeds, the connectivity maps derived from left and right ROI were averaged to create a single connectivity map, consistent with a prior study [16].

For the visual network, we selected two allelic seeds located approximately in the bilateral V2 primary cortex, which had been used in previous studies for mapping visual networks [35], [55]. The two seeds are centered on MNI coordinates (19, −95, 2) and (−19, −95, 2) with a 6-mm radius. The mean time course was extracted and analyzed using correlation analysis.

One-sample *t* tests were performed on the individual z-maps to determine initial intra-group level network regions; the threshold was set at AlphaSim corrected *p*<0.01, cluster size>97. Considering the representation of other nodes within each RSN and the bias of the pre-defined seed approach, a dual regression process was performed (http://fsl.fmrib.ox.ac.uk/fsl/fslwiki/DualRegression). The dual regression process includes the following steps: 1) the initial group spatial maps are regressed into each subject's 4D dataset to give a set of time courses; 2) those time courses are regressed into the same 4D dataset to obtain a subject-specific set of spatial maps; 3) then, within-group and between-group analyses are performed based on the spatial maps acquired in the prior step. Dual regression has been applied in many brain network research studies [56]–[58]. Using dual regression, Wang et al. discovered that the network-wise temporal patterns for the majority of the RSNs (especially for the DMN) exhibited moderate-to-high test-retest reliability and reproducibility under different scan conditions [56]. For within-group statistics, a threshold adjustment method based on the Monte-Carlo simulation correction was used with voxel wise *p*<0.8×10^−5^, cluster size>102 (2754 mm^3^), and cluster connectivity criterion 4 rmm; this outcome yielded an AlphaSim correction threshold of *p*<0.005. Before the group comparison of each map, the within-group threshold maps were combined across the young and elderly groups to create unified masks, which were then used to restrict the between-group analysis. The between-group two sample *t* test analysis was performed at an AlphaSim correction threshold of *p*<0.01, cluster size>97 (2619 mm^3^), and cluster connectivity criterion 4 rmm based on Monte-Carlo simulation correction. The individual modulated gray matter volumes were entered as covariates to regress out the nuisance from the brain volume differences between groups.

## Results

### Within-group analyses of brain networks

**Figure 1:**
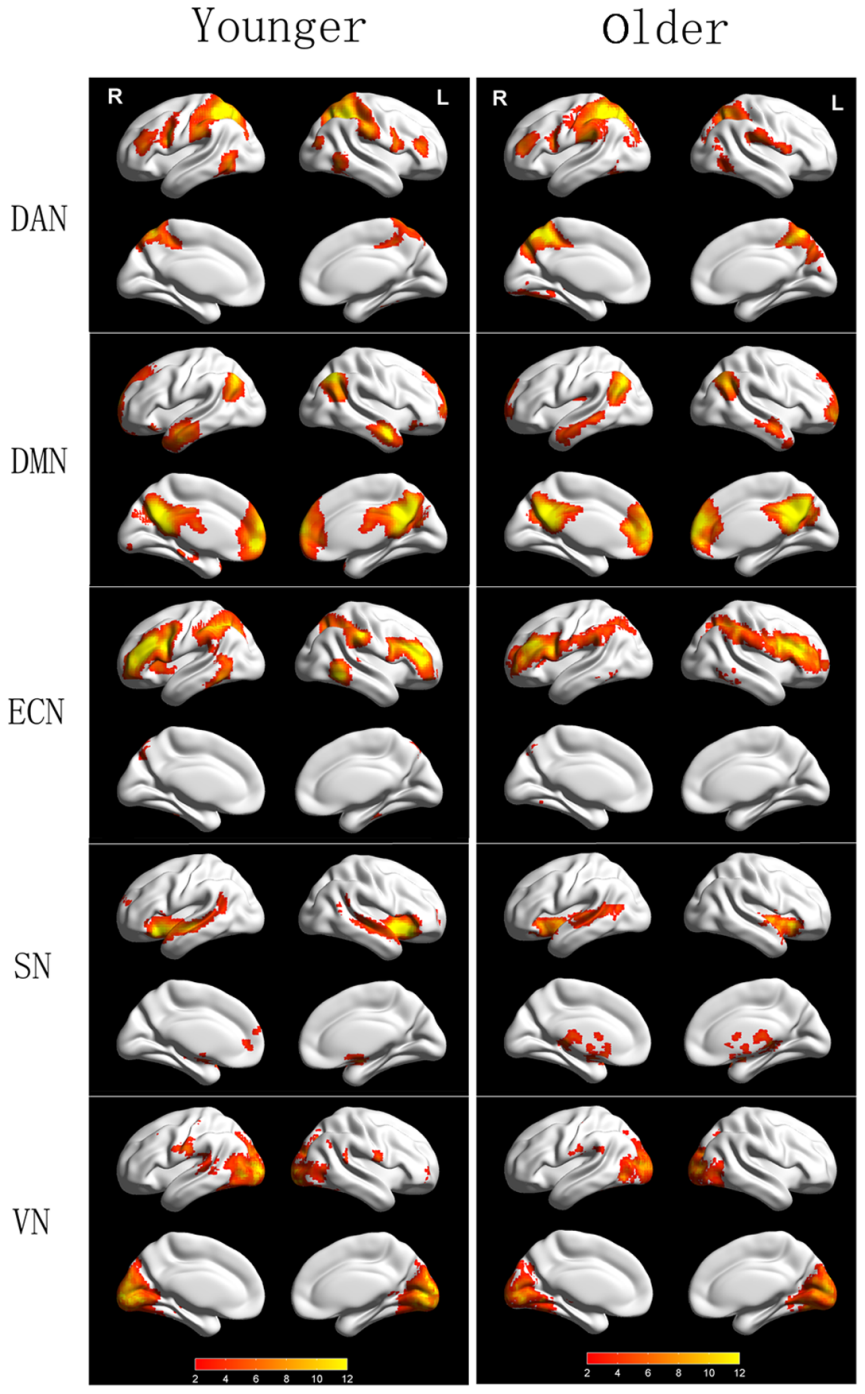
Intra-group maps of canonical networks in the resting brains of younger and older groups. DAN, dorsal attention network; DMN, default mode network; ECN, executive control network; SN: salience network; VN, visual network; R, right view; L, left view. The color bar denotes the T value. The statistical threshold was set at *p*<0.005 and was corrected with AlphaSim.

As shown in Figure 1, the DMN consisted of the PCC, ventral medial prefrontal cortex (vmPFC), bilateral medial temporal cortex (MTC), hippocampus, pulvinars, precuneus and inferior parietal cortex. The regions of the bilateral insular cortex, dorsolateral prefrontal cortex (dlPFC), inferior parietal cortex and posterior middle temporal (area MT+) were involved in the ECN. The DAN consisted of the SPL/intraparietal sulcus (IPS), frontal eye fields (FEF), and extra-striate visual areas, and the SN included the orbital frontal-insular cortex, anterior cingulated cortex, superior temporal pole and subcortical paralimbic regions. The visual network spread across the bilateral primary and association visual cortex. A visual inspection of the RSNs indicated that the connectivity maps for both groups were similar and consistent with prior findings.

### Between-group analyses of brain networks

Distinct alterations were noted in the four canonical networks between the younger and older groups when compared at the same statistical threshold level. In other words, the ECN was disrupted mainly in the older group, followed by the DAN; the DMN and SN showed limited functional connectivity disruption relative to the ECN and DAN.

**Figure 2:**
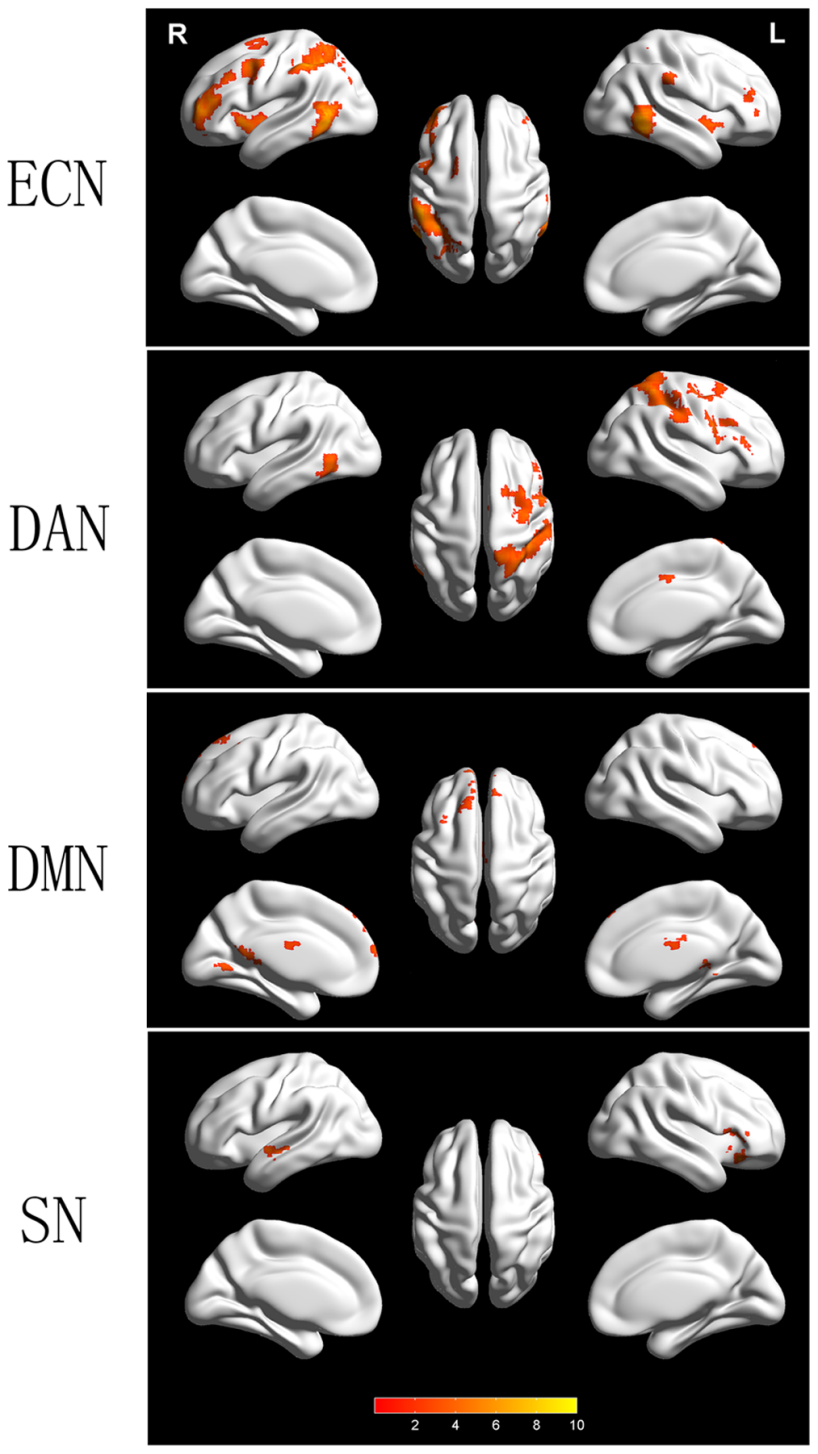
Comparisons of canonical networks between the younger and older groups. The visual network did not exhibit significant differences and is not shown on the map. The right and left lateralized disruptions are shown in the ECN and DAN, respectively. The threshold was set at *p*<0.01 and was corrected with AlphaSim. ECN, executive control network; DAN, dorsal attention network; DMN, default mode network; SN, salience network; R, right view; L, left view. The color bar denotes the T value.

For the ECN, 10 clusters (total of 2,668 voxels, 72,036 mm^3^) presented significantly decreased connectivity in the older group, including regions of the bilateral dorsal frontal cortex, insular cortex, MT+ and right inferior parietal lobe, with a rightward hemisphere asymmetry. In the DAN, 6 clusters (total of 1,400 voxels, 37,800 mm^3^) displayed reduced connectivity in the areas of the parietal postcentral gyrus, supplementary motor area and left mid-occipital gyrus, with a leftward asymmetry. For the DMN, 4 clusters (total of 612 voxels, 16,524 mm^3^) involving the bilateral regions of vMPFC, PCC, pulvinars and bilateral dorsal MPFC presented significantly reduced connectivity. The SN showed the least connectivity reduction in the regions of the left frontal-insular cortex and right superior temporal cortex, with 3 clusters (310 voxels, 8,370 mm^3^) (see Figure 2, details in Table 1). Significantly increased connectivity was not detected in any of the canonical networks in the older group.

**Table 1:** Primary clusters showing significant connectivity alterations in resting-state networks in younger and older groups.

Regions	Peak T	MNI Coordinates			Brodmann area
		x	y	z	
**DMN**					
bilateral MPFC	7.77	0	63	21	10,9,8
PCC	4.92	−3	−55	′9	30
left superior frontal gyrus	3.59	−27	21	54	6
right midfrontal gyrus	5.73	30	26	51	8
right superior frontal gyrus	3.97	11	43	51	9
**DAN**					
right midtemporal gyrus	4.78	54	−60	−9	37,19
left supramarginal gyrus	5.02	−60	−18	36	40
left postcentral gyrus	5.33	−34	−50	63	6,9
left inferior frontal gyrus	6.26	−59	14	26	46,9
left superior frontal gyrus	3.69	−23	12	63	8
supplementary motor area	4.39	9	3	45	24,6
**ECN**					
right MT+	4.68	63	−51	0	37,21
left MT+	4.99	−54	−57	3	37
right midfrontal gyrus	6.16	43	50	10	44,45,48
left midfrontal gyrus	4.34	−57	18	31	44
right inferior lobe	6.06	48	−49	53	40
left superior lobe	5.12	−45	44	59	40
right precentral gyrus	4.84	50	10	43	6,44
left supramarginal gyrus	3.93	−62	−26	35	2
**Salience Network**					
left frontal-insular gyrus	4.19	−33	24	−15	47
right superior temporal gyrus	3.41	56	−3	−8	22

In the control analysis, the visual networks did not demonstrate significant connectivity differences between the two experimental groups.

**Figure 3:**
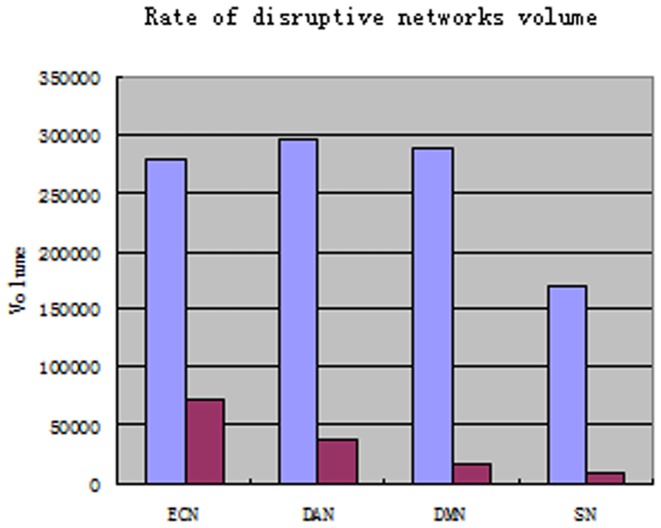
Histogram of the ratio of the disruptive network volume. The light blue bars represent each of the unified mask volumes in the canonical networks; the dark pink bars represent each of the disruptive network volumes in the canonical networks. In the older group, the network volumes were reduced by 25.7%, 12.7%, 5.7% and 4.9% compared with each unified mask for the DMN, DAN, ECN and SN, respectively. This histogram reflects the distinct influence of aging on the canonical networks.

Considering that the canonical networks have different scales, the volume of connectivity decrease for each network was compared with the volume of each combined mask of the paired networks for the two groups (ratio of disruptive network volume) (Figure 3). For the ECN, DAN, DMN and SN, each disruptive network volume accounted for 25.7%, 12.7%, 5.7% and 4.9%, respectively, in each unified mask volume. Although the DAN had the largest volume, it was the second most impaired network in the older group. The map for the ratio of disruptive network volume can reflect the varying degree to which the canonical networks were affected.

**Figure 4:**
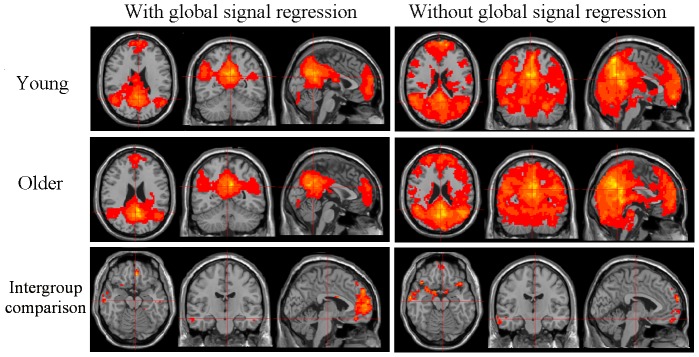
DMN contrasting maps with and without global signal regression for the two experimental groups. “Young” indicates the intra-group analyses with one t test for the younger adults. “Older” indicates the intra-group analyses with one *t* test for the older adults. The threshold for the intragroup and intergroup statistical analyses was set at *p<*0.01 and was corrected by AlphaSim. Note the positively biased correlations and the non-neural noise in the basilar cistern without global signal regression.

Because there are debates concerning global signal regression, which could impact the resting-state fMRI inferences [59] and might reduce the effects of age-related differences on the connectivity in the blood flow, we estimated the results without global signal regression. We observed that the areas involved in each of the canonical networks exceeded the traditional network regions with positively biased correlations for the intra-group analyses, which is consistent with a recent study [60], and we found that the nuisance from the cerebrospinal fluid was incorporated into the maps for the inter-group analyses. DMN contrasting maps with and without global signal regression were used to illustrate the findings (Figure 4). The network maps without global signal regression for the intragroup and intergroup analyses are shown in Figure S1 and Figure S2. We believe that the global signal regression procedure might be appropriate in our study.

## Discussion

Age-related functional brain changes have been extensively explored during tasks and during resting states; however, less is known regarding the effect of normal aging on large-scale brain networks. The present study analyzes the effects of aging on resting-state canonical networks. Our major finding is that selective vulnerability is present in large-scale resting brain networks in normal older adults. The functional connectivity in these canonical networks was selectively reduced as a function of age, even though these networks varied in size.

The most intensive connectivity disruption was demonstrated in the ECN, followed by the DAN and DMN. The salience network was minimally affected, and the visual network, which served as the control, was not significantly affected. These findings are similar to the common observation that age-related processes are characterized by the loss of neural specificity within distinct functional systems [61]. Reduced signal coordination within specific networks may imply less effective functional communication in that specific brain system.

Previous research has shown that clinical neurodegenerative syndromes involving AD, behavioral variant frontotemporal dementia, semantic dementia and progressive nonfluent aphasia each include distinct network vulnerability patterns. For example, the DMN is vulnerable in AD, and the behavioral variant frontotemporal dementia targets the SN [12]–[14]; schizophrenia-vulnerable networks include the ECN and DAN [16]. Our findings suggest that normal aging is also characterized by selective neuronal network vulnerability, with ECN as the first target.

Executive control function, which determines the manipulation and modulation of concrete information processing for many cognitive tasks, is thought to be a higher order cognitive activity that is critically dependent on the ECN. Cognitive tasks require segregated and integrated processing in these large-scale brain networks; for example, the ECN may flexibly couple with the DMN and DAN according to the task domain [5], [10], [62]. Connectivity disruption in the ECN implies that the communication signals between these higher order brain networks have been impaired. Neurobehavioral tests have suggested that normal aging is accompanied by a decline in a range of cognitive abilities that are thought to rely on executive functioning and that executive skills seem to be particularly vulnerable to the effects of aging [43]–[46], [50]. By demonstrating that the ECN is impaired most severely among the canonical brain networks, our findings could provide support for the behavioral theories of cognitive aging.

Several studies have demonstrated the influence of aging on large-scale networks during various task states; for instance, during a semantic classification task, the DMN and DAN are markedly disrupted in advanced aging [35]. Grady et al. measured the connectivity within the DMN and task-positive networks during multiple cognitive tasks [63]. These authors noted that the functional connectivity in the DMN was reduced in older adults, whereas the pattern of task-positive network connectivity was equivalent in younger and older groups [63]. Task-directed age-comparative fMRI investigations showed that older adults have decreased and increased brain activation patterns and that these patterns can shift depending on the degree of the task demand [64]–[66]. Although the aging brain preserves the adaptive function for supporting explicit tasks, our findings indicated that the underlying brain networks were disrupted to varying degrees. Therefore, studying the aging brain using resting-state network connectivity measurements may be advantageous.

The DMN participates in episodic memory, presenting nonspecific deactivation during externally directed tasks. The DMN is active when individuals are engaged in internally focused tasks, including retrieving autobiographical memory, envisioning the future, and conceiving the perspectives of others [3], [9]–[11], [67], [68]. Disruption of the DMN implies impairment in these associated functions. Based on our findings, the DMN was mildly disrupted relative to the ECN and DAN, even though the DMN was sensitive to aging, as previously documented.

Currently, there are two models of hemispheric asymmetry about aging brain: the right hemi-aging model and the hemispheric asymmetry reduction in older adults (HAROLD) model. The former model, which proposes that the right hemisphere shows greater age-related decline than the left hemisphere, is supported by behavioral studies in the domains of cognitive, affective, and sensorimotor processing; the latter model proposes that the prefrontal activity during cognitive performance, which tends to be less left-lateralized in older adults than in younger adults, is supported by evidence from task-directed fMRI [69], [70]. In terms of our present observations on the asymmetric distribution patterns of the ECN and DAN, the two models are not incompatible; e.g., the right lateralized disruption in the ECN may support the right hemi-aging model, and the left lateralized disruption in the DAN may support the HAROLD model. The aging brain models could depend on which network resource is more demanding.

The salience network was affected minimally in the older group, with decreased coordination between regions of the left FI and right superior temporal cortex. The salience network is involved in emotion processing, reward and interoceptive regulation [5], [71], [72]. Previous research has suggested that, on average, the emotional well-being of adults is stable and well maintained or even improved as they age, despite the fact that their physical health and cognitive abilities decline with age [73], [74]. Our findings regarding SN changes in the elderly group could support this proposal.

The visual cortex, belonging to a low-order brain system, has broad fiber tracts connecting to the frontal and temporal lobes. Although the higher-order networks exhibit broadly reduced functional cooperation, our results showed that the visual network was spared during the aging process, which is consistent with previous findings [35]. According to supportive anatomical evidence, the occipital visual areas have the highest neuronal density and the most differential cytoarchitectonic structures in all cortices of the brain [75], [76]. Although many studies indicated that the higher visual attentional function in older adults was impaired, our findings may only reflect the primary photic sense function in older adults.

Selective neuronal and molecular vulnerability could account for the selective network vulnerability in the aging brain. A pattern of selective loss of synapses and neurons in certain brain regions has been described during the aging process [77]–[79]. Genomic analyses have suggested that different molecular mechanisms exist in different neural subtypes that determine their survival or vulnerability to death, which is evidenced by several transgenic mouse model subsets [15].

According to our findings, the older group presented decreased resting functional connectivity within multiple networks rather than increased functional connectivity; therefore, we hypothesize that reduced brain functional cooperation could be a critical pattern as aging progresses.

We acknowledge that the present study has several limitations. First, the relationship between the alteration of networks and behavioral performance requires additional investigation. Second, age-related preclinical atherosclerosis risks may have several effects on the brain's functional connectivity; for example, changes in interactions between the amygdala and rostral ACC have been implicated in an increased risk of atherosclerosis [80]. Thus, atherosclerosis co-variations should be quantified in the future. Additionally, the DMN is inhomogeneous and was decomposed into two or three sub-networks in previous studies [81], [82]. The DMN was not further subdivided in our study.

In summary, our results suggest that the process of aging in the brain is characterized by selective vulnerability in large-scale brain systems. Specifically, the ECN, DAN and DMN are among the canonical networks that are sensitive to the effects of aging. Moreover, high-order brain networks are preferentially affected compared to low-order systems. These findings could provide insight to further our understanding of patterns in age-related decline in multiple cognitive functions.

## Supporting Information

Figure S1 **Intra-group maps of canonical networks without global signal regression in the resting brains of younger and older groups.** DAN, dorsal attention network; DMN, default mode network; ECN, executive control network; SN: salience network; VN, visual network; R, right view; L, left view. The color bar denotes the T value. The statistical threshold was set at *p*<0.001 and was corrected with AlphaSim. The map shows that the areas involved in each of the canonical networks exceed the traditional network regions.
(TIF)

Figure S2 **Comparison of the canonical networks between the younger and older groups without global signal regression procedure.** DAN, dorsal attention network; DMN, default mode network; ECN, executive control network; SN: salience network. Left is left. The color bar denotes the T value. The statistical threshold was set at *p*<0.01 and was corrected with AlphaSim. The prominent nuisance from the cerebrospinal fluid, arteries and veins can be noted, particularly on maps of the SN and DMN.
(TIF)
