# Supplementary figures and images for: Selective Vulnerability Related to Aging in Large-Scale Resting Brain Networks
Source: PLoS One. 2014 Oct 1;9(10):e108807. doi: 10.1371/journal.pone.0108807 (PMC4182761; doi:10.1371/journal.pone.0108807)

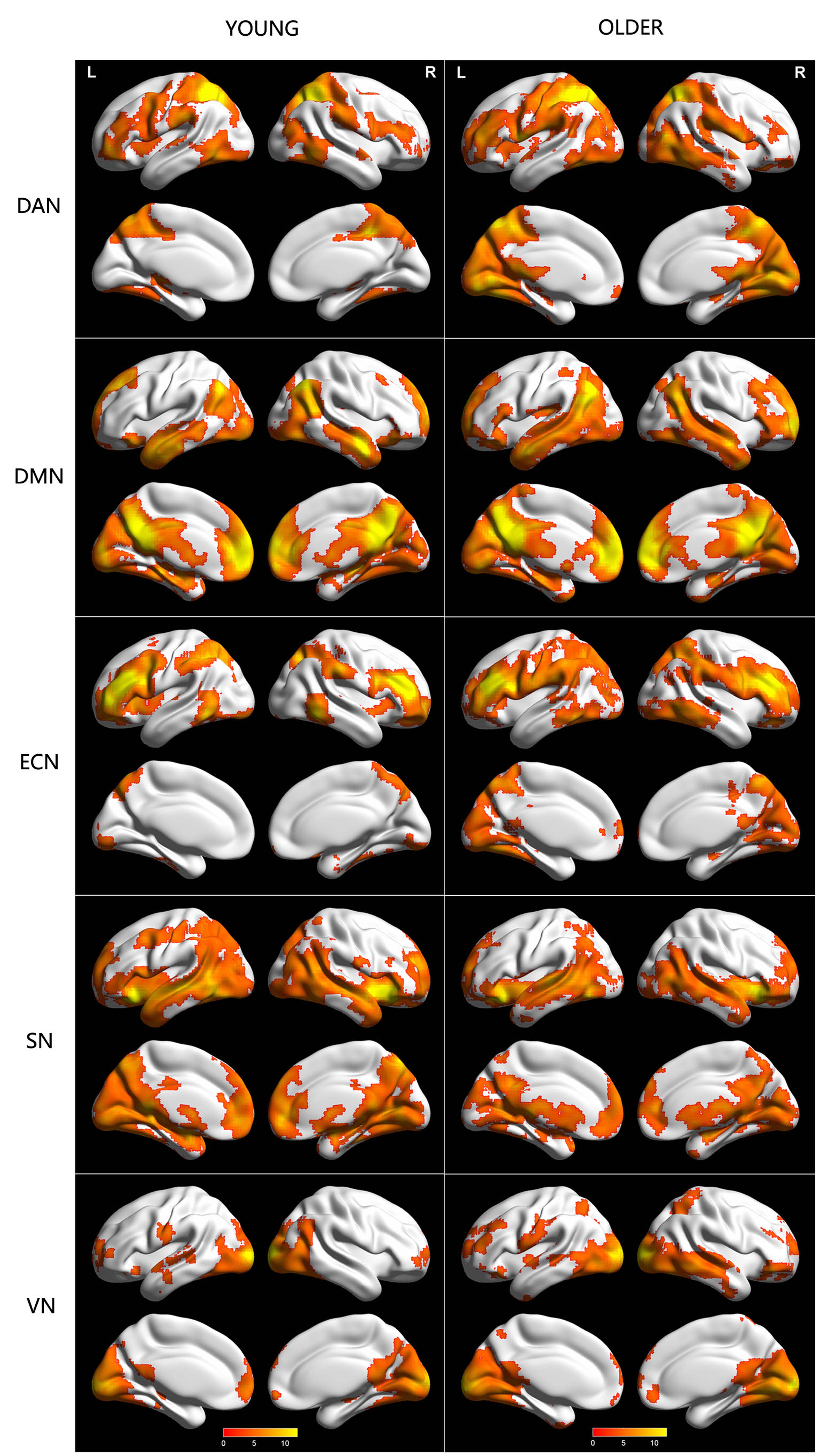

Supplement: Figure S1 — Intra-group maps of canonical networks without global signal regression in the resting brains of younger and older groups. DAN, dorsal attention network; DMN, default mode network; ECN, executive control network; SN: salience network; VN, visual network; R, right view; L, left view. The color bar denotes the T value. The statistical threshold was set at p<0.001 and was corrected with AlphaSim. The map shows that the areas involved in each of the canonical networks exceed the traditional network regions. (TIF) [file pone.0108807.s001.tif]

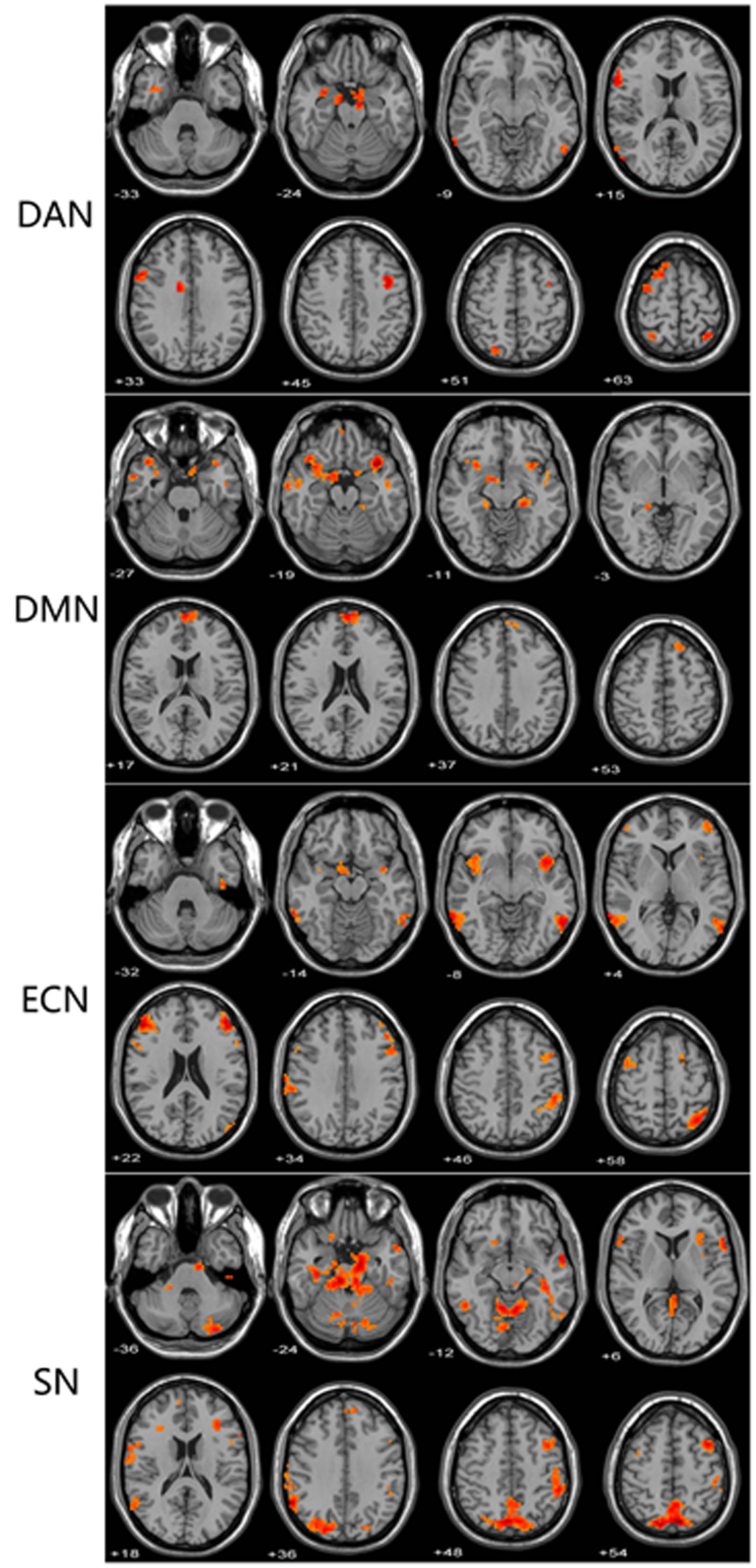

Supplement: Figure S2 — Comparison of the canonical networks between the younger and older groups without global signal regression procedure. DAN, dorsal attention network; DMN, default mode network; ECN, executive control network; SN: salience network. Left is left. The color bar denotes the T value. The statistical threshold was set at p<0.01 and was corrected with AlphaSim. The prominent nuisance from the cerebrospinal fluid, arteries and veins can be noted, particularly on maps of the SN and DMN. (TIF) [file pone.0108807.s002.tif]
